# Periodontitis and bone mineral density among pre and post menopausal women: A comparative study

**DOI:** 10.4103/0972-124X.65434

**Published:** 2010

**Authors:** Snophia Suresh, T. S. S. Kumar, P. K. Saraswathy, K. H. Pani Shankar

**Affiliations:** *Reader, Department of Periodontics, Thaimoogambigai Dental College, Chennai, India*; 1 *Professor and Head, Department of Periodontics, Ragas Dental College, Chennai, Indi*a; 2 *Professor and Head, Department of Periodontics, Matha Dental College, Chennai, India*; 3 *Professor and Head, Department of Periodontics, SRM Dental College, Chennai, India*

**Keywords:** Bone mineral density, osteoporosis, osteopenia, periodontitis

## Abstract

**Aim:**

The aim of the study was to assess the relationship between bone mineral density and periodontitis in premenopausal and postmenopausal women.

**Materials and Methods:**

Twenty women between the age group of 45-55 years were selected for this study. Ten premenopausal women with healthy periodontium constituted the control group and 10 postmenopausal women with ≥2mm of clinical attachment loss in >30% of sites constituted the study group. All patients were assessed for plaque index, probing depth and clinical attachment loss. Radiographs (six IOPA and two posterior bitewing) were taken and assessed for interproximal alveolar bone loss. The patients were scanned to assess the bone mineral density of lumbar spine (L2) and femur using dual energy X-ray absorptiometry (DEXA).

**Results:**

The bone mineral densities of lumbar spine (L2) and femur were significantly lower in the study group than the control group. Osteopenia of the lumbar spine and femur was observed in 60% whereas osteoporosis of lumbar spine was observed in 30% of cases in study group.

**Conclusion:**

Increased proportion of osteopenia and osteoporosis cases of lumbar spine and femur in postmenopausal women with periodontitis suggests that there is association between bone mineral density and periodontitis.

## INTRODUCTION

Osteoporosis and osteopenia are characterized by reduction in bone mass and may lead to skeletal fragility and fracture. Postmenopausal osteoporosis is closely associated with estrogen deficiency[1] that results in increased resorption of bone compared to bone formation. Hence, the fracture of the spine, neck of the femur and radius are the main areas of clinical manifestations of postmenopausal osteoporosis. The periodontal manifestations in menopause include alveolar bone resorption, clinical attachment loss and tooth loss.

The mechanisms by which systemic bone loss may lead to more severe periodontal destruction are decreased local bone mineral density caused by systemic bone loss, altered local tissue response to periodontal infections, genetic factors and changed life style patterns like smoking alcohol consumption etc. These may put an individual at risk for both osteoporosis and periodontal disease.

### Aims and objectives

1. To assess the relationship between bone mineral density and periodontitis in premenopausal (control group) and postmenopausal women (study group).
2. To ascertain that the severity of alveolar bone loss could be a risk indicator for systemic bone loss.
3. To establish that periodontitis may be used as a routine screening procedure for osteoporosis in postmenopausal women.

## MATERIALS AND METHODS

Twenty women aged between 45-55 years were randomly selected from out patients attending the Department of Periodontics, Tamil Nadu Government Dental College and Hospital.

### Inclusion criteria - (for both groups)

1. Age Group 45-55 Years
2. Patients should have at least 15 natural teeth

### Exclusion criteria - (for both groups)

1. Patients who need antibiotic prophylaxis
2. Parathyroid disease
3. Metabolic bone disease
4. Malignancy
5. Early onset of menopause
6. Patients on long term steroid medication, hormone replacement therapy (HRT) and calcium
7. The use of contrast agents or participation in nuclear medicine studies seven days prior to bone mineral density (BMD) assessment.

### Study design

**Figure 1:**
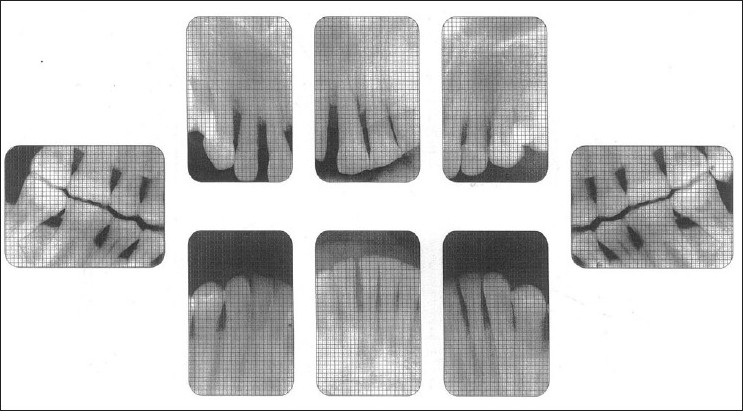
Radiograph of study group with grid

The patients were categorized into two groups, namely, the study and control group. The control group consisted of 10 premenopausal women in the age group of 45-55 years who exhibited healthy periodontium with no attachment loss. The study group consisted of ten postmenopausal women aged between 45-55 years constituted the study group, who exhibited ≥ 2mm clinical attachment loss in > 30% of sites. Informed consent was obtained from all the patients. The following clinical parameters were recorded:

- Plaque index[2]
- Probing depth
- Clinical attachment level
- Interproximal alveolar bone loss was determined from six intra oral periapical radiographs and two posterior bitewing radiographs. The radiographs were scanned and digitized.[3] A grid calibrated in millimeters was superimposed on the radiographs using corel-draw software [Figure 1]. The interproximal alveolar bone loss was measured from cementoenamel junction to most coronal aspect of interproximal alveolar bone on the mesial and distal aspects of all teeth except for 3rd molars.

### Bone mineral density

**Figure 2:**
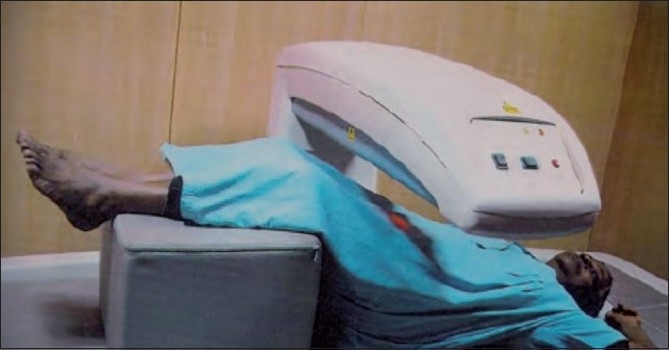
Position of patient to measure AP spine bone density

**Figure 3:**
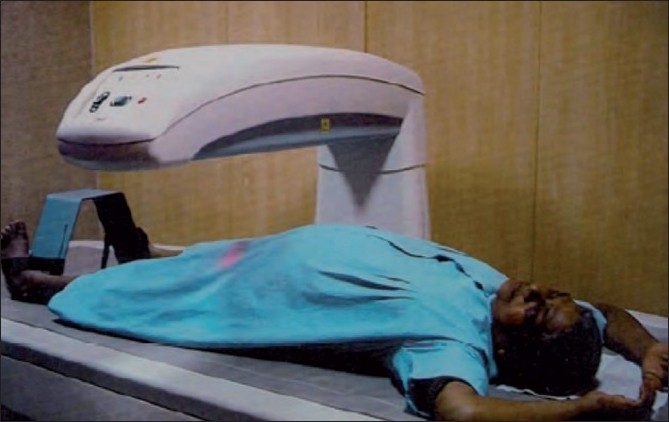
Position of patient to measure femur bone density

**Figure 4:**
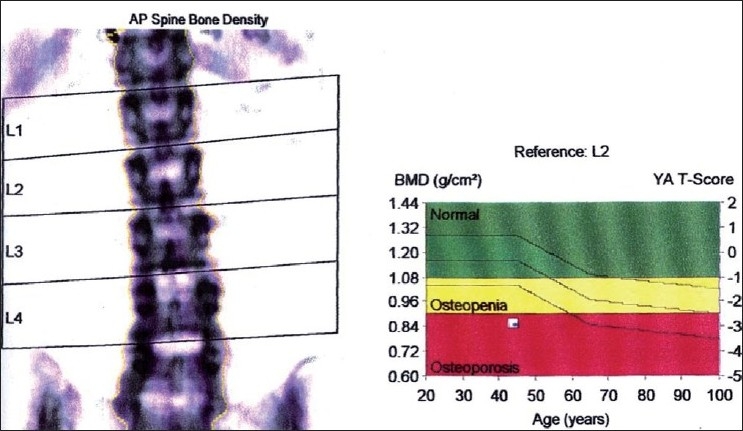
DEXA report - AP spine bone density study group

**Figure 5:**
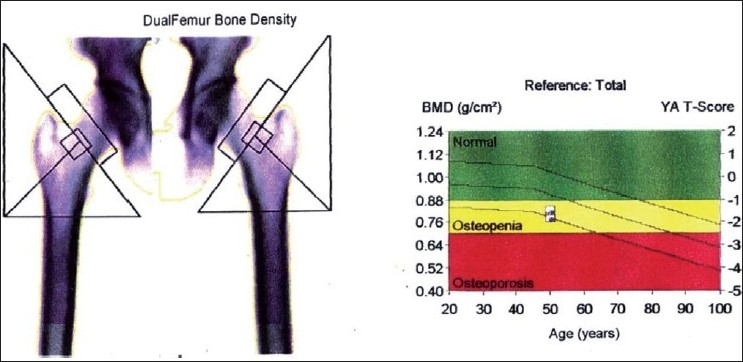
DEXA report - Dual femur bone density study group

The bone mineral density values of anterior-posterior lumbar spine and dual femur were assessed using a dual energy X-ray absorptiometer[4] (DEXA) [Figures 2 and 3]. DEXA is a two dimensional projection system that uses an X-ray tube source as photon source. In this technique X-ray tube emits two X-ray beams which pass through bone are picked up by detector. Computer is used to analyze the resulting images and calculate bone mineral density based on the amount of radiation absorbed by the bone [Figures 4 and 5].

### The readings obtained were

1. Bone mineral density –the bone mineral density of the focused area which is expressed in units of grams/cm^2^
2. T-score- represents the difference from the peak bone mass for the population expressed in standard deviation (S.D).

Formulae for T-Score assessment:

T-score = (BMD-YN)/S.D.

BMD - Bone mineral density of the focused area.

YN - Young normal women around 30 years.

Reports were analyzed and fracture risk indications were determined by using World Health Organization (WHO) diagnostic guidelines[5]

**Table:** 

1.	Normal	BMD not more than-1 standard deviations (SD) below the mean value of peak bone mass in young normal women.
2.	Low bone mass (Osteopenia)	BMD within-1 standard deviations (SD) and - 2.5 standard deviations (s.d.) of the mean value of peak bone mass in young normal women.
3.	Osteoporosis	BMD less than - 2.5 standard deviations (SD) below the mean value of peak bone mass in young normal women.
4.	Established Osteoporosis	BMD less than-2.5 standard deviations (SD) below the mean value of peak bone mass in young normal women and the presence of fractures.

### Statistical analysis

The data so collected were statistically analyzed. The statistical package SPSS PC+ (statistical package for Social Science, version 4.0.1) was used for statistical analysis.

Mean values were compared by student’s independent t-Test. Proportions of T-score abnormality was compared by chi-square test / Fishers Exact test (2-tailed) appropriately

## RESULTS

The parameters used for statistical analysis were age, number of teeth, BMI, plaque index, probing depth, clinical attachment loss, interproximal alveolar bone loss, bone mineral density of lumbar spine and femur.

**Graph 1:**
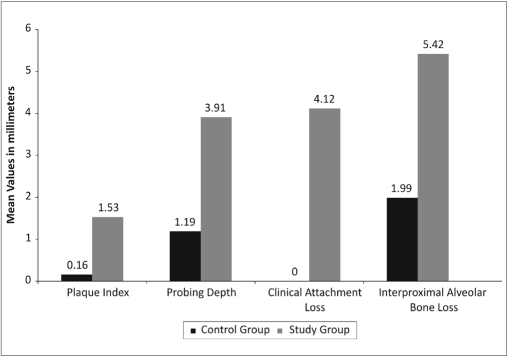
Mean clinical periodontal parameters in control and study group

**Graph 2:**
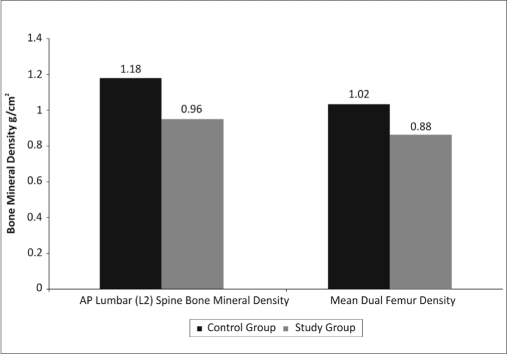
Mean bone mineral densities in control and study group

Tables 1 and 2, Graphs 1 and 2 show the comparison of mean values, standard deviations and *P*-values of clinical parameters of control and study groups respectively.

Table 1 shows the level of significance (*P*<0.0001) of clinical parameters of study group than control group whereas age, BMI and no. of teeth had no significant *P* value. Table 2 shows the bone mineral densities of lumbar spine (*P*<0.0001) and femur (*P*<0.003) which were significantly lower in study group than control group. Table 3 shows the proportion of osteopenia and osteoporosis cases in lumbar spine and femur in control and study groups. There were 60% osteopenia and 30% osteoporosis cases of lumbar spine and 60% osteopenia cases of femur seen in study group.

The results show an increased proportion of osteopenia and osteoporosis cases in lumbar spine and femur in postmenopausal women with periodontitis

**Table 1:** Comparison of mean value, standard deviation and *P*-value between control and study group of clinical periodontal parameters

Parameters	Control group	Study group	*P*-value
	Mean ± S.D.	Mean ± S.D.	
Age	47.1±3.1	49.2±2.7	0.12
Body mass index	24.3±4.1	25.3±4.5	0.61
Number of teeth	29.1±2.2	28.4±2.4	0.51
Plaque index	0.16±0.12	1.53±0.40	<0.0001*
Probing depth (mm)	1.19±0.14	3.91±0.46	<0.0001*
Clinical attachment loss (mm)	0.0±0.0	4.20±0.73	<0.0001*
Interproximal alveolar bone loss (mm)	1.94±0.34	5.42±0.57	<0.0001*

* Significant

**Table 2:** Comparison of mean value, standard deviation and *P*-value between control and study group of bone mineral densities (g/cm^2^) of AP lumbar spine (L_2_) and femur

Parameters	Control group	Study group	*P*-value
	Mean ± S.D.	Mean ± S.D.	
AP lumbar spine (L_2_) (g/cm^2^)	1.18±0.08	0.96±0.07	<0.0001*
Mean dual femur density (g/cm^2^)	1.02±0.05	0.88±0.11	<0.003*

* Significant

**Table 3:** Proportion of normal, osteopenia and osteoporosis cases of AP lumbar spine (L_2_) and dual femur densities in control and study group

Parameters	Control group		Study group		*P*-value
	Mean ± S.D.		Mean ± S.D.		
AP lumbar spine (L_2_)					
Normal	10	10%	1	10%	
Osteopenia	0	0%	6	60%	<0.0001**
Osteoporosis	0	0%	3	30%	
Femur					
Normal	10	100%	4	40%	0.01*
Osteopenia	0	0%	6	60%	

* Significant;

** Highly Significant

## DISCUSSION

Osteoporosis means literally “porous bone,” a condition of there being “too little bone” to provide mechanical support. Osteopenia is a reduction in BMD below a pre defined level. Osteoporosis is characterized by a reduction in bone mineral density to level below what is required for mechanical support. A consensus development conference defined osteoporosis as a systemic skeletal disease characterized by low bone mass and microarchitectual deterioration with a consequent increase in bone fragility and susceptibility to fracture.[6]

Osteoporosis may be a primary disorder or secondary to other diseases or conditions. Primary osteoporosis includes idiopathic and involutional form. Idiopathic forms of osteoporosis are rare and affect men and women equally. Involutional osteoporosis includes two patterns type I (postmenopausal) and type II (age related) osteoporosis.

Type I osteoporosis occurs in postmenopausal women. Bone loss in premenopausal women is slow and approximately equal to that of men (0.3 to 0.5% per year). With the onset of menopause in females, an accelerated rate of cortical bone loss of two to three per cent per year for about eight to ten years. Type I osteoporosis related to estrogen deficiency associated with menopause, leads to a cascade of accelerated bone loss by decreased secretion of parathyroid hormone, increased secretion of calcitonin and decreased calcium absorption which further aggravates bone loss.

In addition, patients with type I osteoporosis and high bone turnover have increased production of IL – 1 from stimulated monocytes as compared with age matched controls, which also contributes to increased bone resorption.[7]

Type II (age-related) osteoporosis appears to affect virtually the entire population of aging men and women.

Periodontitis is an inflammatory disease characterized by loss of connective tissue and alveolar bone. Like osteoporosis, it is a silent disease, not causing symptoms until late in the disease process when mobile teeth, abscesses and tooth loss may occur. While the etiologic agent in periodontitis is a pathogenic bacterial plaque in a susceptible patient, periodontitis and osteoporosis have several risk factors in common. They include an increased prevalence with age, smoking and influence of disease or medications that may interfere with healing.

The possible mechanism by which postmenopausal osteoporosis lead to more periodontal destruction may be the presence of less crestal alveolar bone per unit volume, this bone of lesser density may be more easily absorbed. Estrogen acts by blocking the production of cytokines that promote osteoclast differentiation and osteoclast apoptosis.

Estrogen withdrawal following menopause is associated with increased osteoclast numbers due to enhanced osteoclast formation and activity and reduced osteoclast apoptosis. Elevated IL-1 and IL-6 and TNF-α induce osteoclast activity and increased bone turnover rates and finally the systemic factors of bone remodeling may also modify local tissue response to periodontal infection.[8] Persons who have systemic bone loss may react to periodontitis with increased production of cytokines and inflammatory mediators.

In an earlier study by Wactawski Wende *et al*,[9] conducted in Caucasian women, the ages of postmenopausal women selected for the study were between 51 to 78 years. In Indian women, menopause occurs between the ages of 45 and 55; the patients selected for this study were between the age group 45 - 55 years.

The risk factors for osteoporosis as given by WHO study group 1994[5] include low BMI, premature menopause, family history of osteoporosis, patients on thyroid, anti convulsant and steroid medications were excluded in the study.

In a previous study,[10] X-ray examination of the vertebral column was done for diagnosis of osteoporosis. In the present study dual energy X-ray absorptiometry (DEXA) was used to assess bone mineral density in lumbar spine and femur which is similar to studies by Reinhart *et al*.[11] Dual energy X-ray absorptiometry (DEXA) measurements are good predictors of fracture risk. The average radiation exposure for DEXA is 1 to 3 mrad, per scan and it is a safe method for assessing bone mineral density. In an earlier study,[1] the metacarpal index was used to detect osteoporosis, in the present study bone mineral density in anterior posterior view of lumbar spine and femur were assessed in concurrence to study by Wactawski Wende *et al*.[12]

Femoral neck and vertebral fractures are related to postmenopausal osteoporosis. Bone mineral density of lumbar spine (L2) was used for analysis in the present study because it is the vertebrae least affected by artifacts. The assessment of alveolar process density in dentate subjects is limited anatomically to small inter radicular regions preclude the use of DEXA.

Regions of bone that consist predominantly of trabecular structure like vertebrae are the preferred sites for the assessment of bone mineral density because of earlier detection of change in bone mineral content.[13]

Numerous investigators utilized dental radiographs to compare the alveolar bone loss with the bone mineral density of skeletal sites.[14–16] In the present study, interproximal alveolar bone loss was measured from the cementoenamel junction to the most coronal aspect of the interproximal alveolar bone for each tooth on the mesial and distal side from the radiographs, which is similar to the studies by Hausman *et al*,[17]. Interproximal bone loss >2mm was considered as suggested in a study by Albondar *et al*.[19] The measurements were not made in third molars similar to study by Hildebolt *et al*,[18] because of variations in the position of the 3rd molars.

As far as the dental parameters are concerned, statistically, the probing depth in the study group was significantly higher (*P*<0.0001) than the control group and not in concurrence with previous study.[9]

Statistically, bone mineral density showed significantly lower value for the lumbar spine (*P*<0.0001) and dual femur (*P*<0.003) in the study group than control group. The mean interproximal alveolar bone loss is higher (5.42 mm) than mean probing depth (3.91mm) and clinical attachment loss (4.20mms) in the study group. This implies the importance of indirect systemic effect of osteopenia on periodontal disease as stated by Wactawski Wende.[9]

The proportion of osteopenia and osteoporosis cases in this study were 100% normal cases in control group and 10% normal, 60% osteopenia and 30% osteoporosis cases in study group as far as the lumbar spine (L2) density is concerned; and 100% normal in control group and 40% normal and 60% osteopenia cases in the study in the study group, as far as dual femur density is concerned which is not observed in the previous study.[9]

The incidence of osteopenia and osteoporosis in percentage calculation is significantly higher in lumbar spine (L2) (60% osteopenia, 30% osteoporosis) when compared to dual femur (60% osteopenia). This shows that the trabecular bone is found in greater amounts in the vertebral bodies compared to ends of long bones. The trabecular bone in postmenopausal woman is lost earlier and more rapidly than cortical bone due to estrogen deficiency.[20]

The periodontal clinical parameters such as probing depth, clinical attachment loss and interproximal alveolar bone loss were significantly higher (*P*<0.001) in study group compared to control group and the bone mineral density is significantly lower in study group (*P*<0.001 and *P*<0.003) compared to control group. Based on these findings, the present study, proved positively the association of periodontitis with systemic bone mineral density in post menopausal women.

## CONCLUSIONS

The occurrence of osteopenia and osteoporosis of the lumbar spine (L2) and femur in postmenopausal women with periodontitis suggests that there is association between bone mineral density and periodontitis and that the severity and extent of alveolar bone loss in postmenopausal women may be a risk indicator for systemic bone loss. Routine periodontal screening will go a long way in the early detection of bone changes, disease status and treatment modalities, so compulsory referral from the medical side to the periodontist for the status of the alveolar bone in post menopausal women is a must.
